# Antifungal Activity of Genistein Against Phytopathogenic Fungi *Valsa mali* Through ROS-Mediated Lipid Peroxidation

**DOI:** 10.3390/plants14010120

**Published:** 2025-01-03

**Authors:** Fangjie Li, Chen Yang, Maoye Li, Su Liu, Kuo Xu, Xianjun Fu

**Affiliations:** 1 Research Institute for Marine Traditional Chinese Medicine (Qingdao Academy of Chinese Medical Sciences), The SATCM’s Key Unit of Discovering and Developing New Marine TCM Drugs, Key Laboratory of Marine Traditional Chinese Medicine in Shandong Universities, Shandong University of Traditional Chinese Medicine, Jinan 250355, China; lifj819007@163.com; 2 Shandong University of Traditional Chinese Medicine Qingdao Academy of Chinese Medical Sciences, Qingdao Key Laboratory of Research in Marine Traditional Chinese Medicine, Qingdao Key Technology Innovation Center of Marine Traditional Chinese Medicine’s Deep Development and Industrialization, Qingdao 266114, China; 3 Anhui Provincial Key Laboratory of Integrated Pest Management on Crops, School of Plant Protection, Anhui Agricultural University, Hefei 230036, China; chenyang199874@163.com (C.Y.); limaoye81@ahau.edu.cn (M.L.); suliu@ahau.edu.cn (S.L.)

**Keywords:** *Valsa mali*, phytopathogenic, fungi, pathogenic, genistein

## Abstract

*Valsa mali* (*V. mali*) is a necrotrophic fungus responsible for apple Valsa canker, which significantly diminishes apple production yields and quality in China. Our serendipitous findings revealed that genistein significantly inhibits the mycelial growth of *V. mali*, with an inhibition rate reaching 42.36 ± 3.22% at a concentration of 10 µg/mL. Scanning electron microscopy analysis revealed that genistein caused significant changes in the structure of *V. mali*, including mycelial contraction, distortion, deformity, collapse, and irregular protrusions. Transmission electron microscopy analysis revealed leakage of cellular contents, blurred cell walls, ruptured membranes, and organelle abnormalities. Genistein has been shown to increase reactive oxygen species levels in *V. mali* mycelia, as demonstrated by 2′,7′-dichlorofluorescin diacetate staining. This increase was associated with a decrease in superoxide dismutase activity alongside increases in catalase and peroxidase activities. These changes collectively disrupted the oxidative equilibrium, leading to the induction of oxidative stress. The transcriptomic analysis revealed 13 genes enriched in this process, linked to unsaturated fatty acid biosynthesis (three downregulated DEGs), saturated fatty acid biosynthesis (three upregulated and six downregulated DEGs), and fatty acid metabolism (four upregulated and nine downregulated DEGs). Additionally, the downregulated DEGs VMIG_07417 and VMIG_08675, which are linked to ergosterol biosynthesis, indicate possible changes in membrane composition. In conjunction with the qRT-PCR results, it is hypothesized that genistein exerts an antifungal effect on *V. mali* through ROS-mediated lipid peroxidation. This finding has the potential to contribute to the development of novel biological control agents for industrial crops.

## 1. Introduction

Phytopathogenic fungi pose a significant threat to global food security, ecosystem services, and human well-being [1]. These fungi typically persist through parasitic and saprophytic mechanisms, often resulting in diseases in their host plants. Currently, crops such as apples, tomatoes, and rice are afflicted by fungal diseases caused by species including *Valsa mali* (*V. mali*) [2], *Botrytis cinerea* [3], and *Ustilaginoidea virens* [4]. Among them, *V. mali* is a necrotrophic fungus responsible for apple Valsa canker (AVC), a disease that adversely affects various parts of the apple tree, including the fruit, primary branches, and trunk. This pathogen not only reduces apple yield and quality but can also result in tree mortality and orchard devastation under severe conditions, thereby inflicting substantial losses on apple production [5]. The disease is characterized by its extensive geographical distribution, severe impact, and considerable challenges in prevention and control, earning it the moniker “cancer” of apple trees.

Several strategies have been developed for the management of *V. mali*, including the application of fungicides, excision of cankers, and pruning of necrotic or weakened branches. Benzimidazole fungicides, such as tebuconazole and thiophanate-methyl, are the most frequently employed agents for controlling *V. mali* [6,7]. Nevertheless, the increased frequency and concentration of fungicide application have inadvertently contributed to the development of pathogen resistance and have raised concerns regarding chemical residues, which pose potential risks to human health and the environment. Furthermore, once the pathogen has extensively infiltrated the phloem and xylem of apple trees, its management becomes exceedingly challenging. In conclusion, there is presently no effective and safe pesticide available for the prevention and control of *V. mali*.

The discovery of highly effective and low-toxicity natural antifungal compounds against the phytopathogenic fungus *V. mali* is of considerable importance. Researchers have conducted extensive and commendable investigations in this domain. For example, in vitro experimental findings demonstrated that 6-methylcoumarin, at a concentration of 400 mg/L, achieved an inhibition rate of 94.6% against *V. mali* [8]. Subsequent experiments revealed that 6-methylcoumarin could impede the growth of *V. mali* mycelium and spore germination, leading to damage of the cell membrane. In the inoculated apple branches, 6-methylcoumarin was observed to effectively inhibit the progression of AVC and decrease its incidence rate. Duan et al. conducted an investigation into the bactericidal activity of various tobacco organ samples, revealing that flowers exhibit greater antibacterial activity against apple rot pathogens compared to leaves [9]. Our research group unexpectedly identified that genistein, a widely occurring natural compound, exhibits a significant inhibitory effect on *V. mali*. This study is the first to document the antifungal activity of genistein against the phytopathogenic fungus *V. mali*, mediated through reactive oxygen species (ROS)-induced lipid peroxidation.

In this study, the micro double-dilution method was employed to determine the minimum inhibitory concentration (MIC) of genistein. Additionally, scanning electron microscopy (SEM) and transmission electron microscopy (TEM) were utilized to examine the morphology and ultrastructure of *V. mali* hyphae. To elucidate the antifungal mechanism of genistein, we employed 2′,7′-dichlorofluorescin diacetate (DCFH-DA) staining, assessed the activities of antioxidant protective enzymes, and utilized RNA-sequencing technology to explore the potential inhibitory mechanisms of genistein against *V. mali*. This study represents the first investigation into the inhibitory effects and molecular mechanisms of genistein on the plant-derived fungus *V. mali.*

## 2. Results

### 2.1. Inhibitory Effect of Genistein on the Growth of V. mali

**Figure 1:**
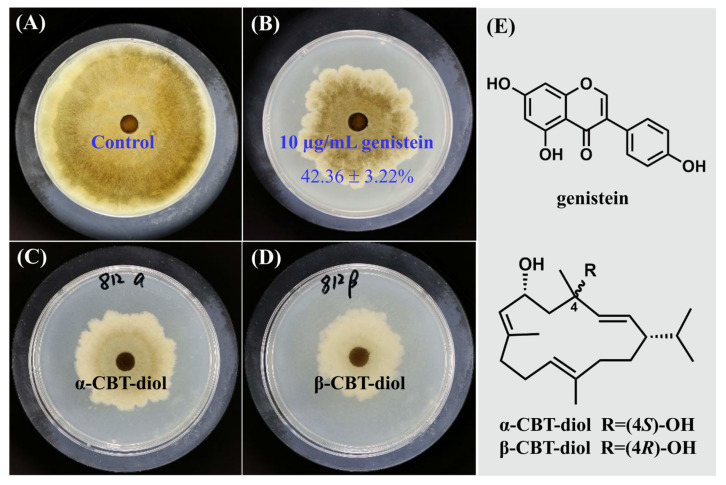
The inhibitory effect of genistein on *V. mali*. (**A**) Blank control; (**B**) Mycelium treated with 10 µg/mL genistein; (**C**) Mycelium treated with 10 µg/mL α-CBT-diol; (**D**) Mycelium treated with 10 µg/mL β-CBT-diol; (**E**) Structures of genistein, α-CBT-diol and β-CBT-diol.

In our study, different concentrations of genistein were prepared using the double-dilution method with anhydrous ethanol as the solvent, and the MIC values of genistein against *V. mali* were determined using the plate method. The results showed that as the concentration of genistein increased, the mycelial inhibition rate of *V. mali* continued to increase. When the concentration of genistein reached 10 µg/mL, no precipitation occurred in the culture medium, and the inhibition rate of genistein on *V. mali* reached 42.36 ± 3.22%, which was close to the inhibition activities of the positive control groups, α-CBT diol (48.03 ± 6.44%) and β-CBT diol (55.99 ± 3.60%) (Figure 1). Therefore, the MIC of genistein against *V. mali* was 10 µg/mL, which was used for subsequent experiments.

### 2.2. Effects of Genistein on the Morphology and Ultrastructure of V. mali Mycelia

**Figure 2:**
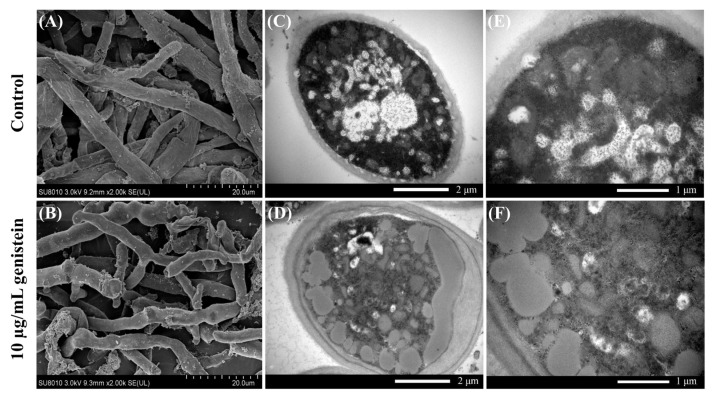
Effects of genistein on hyphal morphology ((**A**,**B**), 2000× magnification) and ultrastructure ((**C**,**D**), 20,000× magnification; (**E**,**F**), 40,000× magnification) of *V. mali*.

To further clarify the damage caused by genistein to *V. mali*, SEM and TEM analyses were performed to observe the morphology and ultrastructure of the hyphae. SEM analysis of the preprocessed *V. mali* mycelia revealed that genistein significantly affected the morphology of the mycelia. The mycelia without genistein treatment exhibited normal linear and cylindrical morphologies with a uniform thickness and smooth surface (Figure 2A). Mycelia treated with 10 µg/mL genistein exhibited significant shrinkage, distortion, deformation, and even collapse, losing their cylindrical shape, which was accompanied by irregular protuberances (Figure 2B).

Under TEM, the mycelia of the control group were neat in cross-section, uniform in thickness, and clear and smooth in terms of structure (Figure 2C,E). Organelles, such as the endoplasmic reticulum and mitochondria, were intact and evenly distributed in the cytoplasm. In contrast, the cytoplasm of the hyphae in the treatment group presented pronounced cytoplasmic fluid transformation and an increase in the number of intracellular cavities (Figure 2D,F). Notably, significant extravasation of the cellular contents, a blurry appearance of the cell walls, rupture of the cell membranes, and abnormalities in organelles were observed. These observations suggest that the cellular structure of the hyphae was severely damaged, indicating the significant antifungal effect of genistein on *V. mali* [10].

### 2.3. Effects of Genistein on the Reactive Oxygen Species (ROS) Level of V. mali

**Figure 3:**
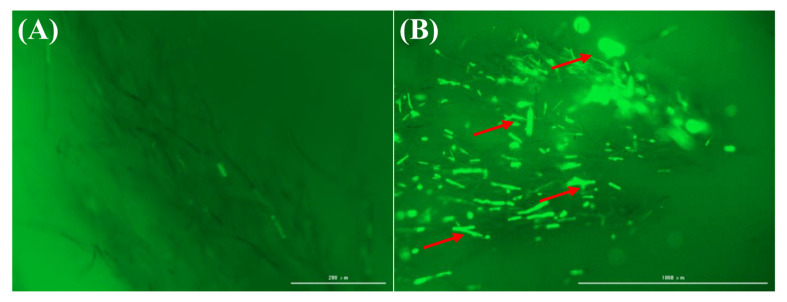
The effect of genistein on the production of reactive oxygen species. (**A**) Blank control; (**B**) Mycelium treated with 10 µg/mL genistein.

The dynamic balance between oxidation and antioxidation reactions in fungal cells is maintained to ensure normal metabolic processes. Once the balance between the two is disrupted, the permeability of the cell membrane increases, causing cell damage [11]. To further explore the mechanism of genistein against *V. mali*, we determined the content of ROS in the mycelium before and after treatment with genistein. After the mycelium was treated with genistein, the mycelium in the control group was almost completely black in the field of view, making it difficult to detect fluorescence. In contrast, more obvious green fluorescence was detected in the treatment group (Figure 3A,B). The strength of the fluorescence signal reflects the intracellular ROS level of the mycelium. These findings suggest that genistein induces the production of ROS in the mycelium, causing oxidative stress damage to the mycelium. The sudden increase in the reactive oxygen species content within the mycelium is an important indicator of lipid peroxidation in biological membranes. Therefore, in the subsequent transcriptome data analysis, we focused on the suppression of hyphal growth resulting from lipid peroxidation triggered by genistein.

### 2.4. Determination of Antioxidant Enzyme Activity

**Figure 4:**
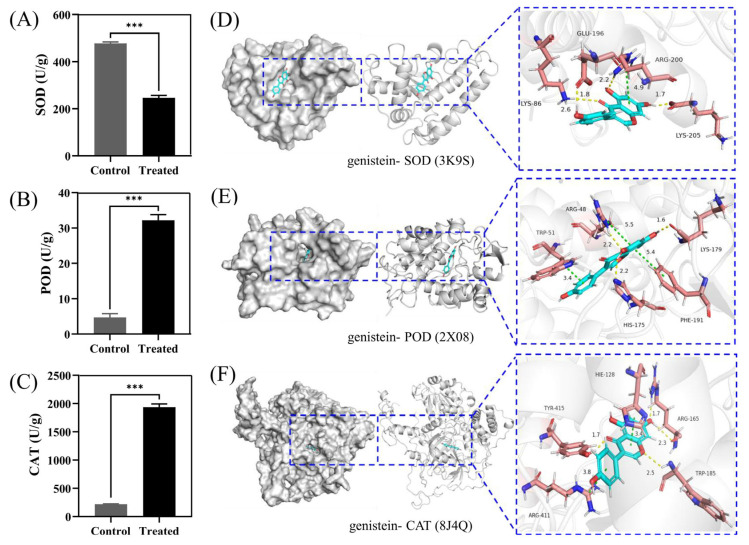
The effect of genistein treatment on the antioxidant enzyme of *V. mali*. (**A**–**C**) Activity testing of SOD, POD, and CAT enzymes. *** *p* < 0.001. (**D**–**F**) 3D maps of docking genistein with SOD, POD, and CAT.

Research has shown that superoxide dismutase (SOD), catalase (CAT), and peroxidase (POD) are the main protective enzymes in fungi, which resist oxidative stress damage to tissues by clearing reactive oxygen species in the fungal body [12]. In this study, after treatment with genistein, the activity of the antioxidant protective enzyme SOD decreased (Figure 4A), whereas the activities of POD (Figure 4B) and CAT increased (Figure 4C), suggesting the activation of the ROS scavenging system in the treatment group [13].

Subsequently, we molecularly docked the proteins and genistein to study their binding patterns and interactions. As shown, proteins are represented by a gray surface and cartoon. Genistein is shown as cyan sticks. The key residues are shown as brick red sticks. Through docking, we found that genistein and three proteins had excellent binding energy (docking score: the smaller the value, the stronger the binding energy. Generally, a score less than −5 indicates strong binding energy), which confirms the above results. In addition, we studied the binding patterns and interactions between the compound and proteins. For SOD protein, the binding energy is −9.399 kcal/mol. Genistein could form four hydrogen bonds and one Pi-Pi interaction with amino acids on the protein, respectively. Specifically, the carbonyl oxygen atom, hydroxyl oxygen atom, and hydroxyl H atom on the skeleton core can form three hydrogen bonds with NH on LYS-86, NH on ARG-200, and carboxylic acid oxygen atom on LYS-205 at distances of 2.6, 2.2, and 1.7 Å, respectively. The H atom on the hydroxy group of phenylcyclone can form a hydrogen bond with the carbonyl oxygen atom on GLU-196 at a distance of 1.8 angstroms. In addition, the skeleton core can also form a Pi-Pi interaction with ARG-200 at a distance of 4.9 angstroms (Figure 4D). For POD protein, the binding energy is −8.727 kcal/mol. The compound can form three hydrogen bonds and three Pi-Pi interactions with amino acids on the protein, respectively. Specifically, the two hydroxyl H atoms and hydroxyl oxygen atoms on the skeleton core can form three hydrogen bonds with the carbonyl oxygen atom on LYS-179, the N atom on HIS-175, and the N atom on ARG-48 at distances of 1.6, 2.2, and 2.2 Å, respectively. In addition, the benzene ring of the skeleton core and side chain can also form two Pi-Pi interactions with ARG-48, Ph-191, and TRP-51 at distances of 5.5, 5.4, and 3.4 angstroms, respectively (Figure 4E). For CAT proteins, the binding energy is −8.350 kcal/mol. The compound can form four hydrogen bonds and two Pi-Pi interactions with amino acids on the protein, respectively. Specifically, the hydroxyl H atom, hydroxyl oxygen atom, carbonyl oxygen atom, and the O atom on the opposite side of the carbonyl group can form four hydrogen bonds with the NH and O atom on ARG-165, the hydroxyl H atom on TYR-415, and the oxygen atom on TRP-185, respectively, at distances of 1.7, 2.3, 1.7, and 2.5 angstroms. In addition, the benzene ring of the skeleton core and side chain can also form two Pi-Pi interactions with HIS-128 and ARG-411 at distances of 3.4 and 3.8 angstroms, respectively (Figure 4F).

### 2.5. Quality Assessment of Sequencing Data

**Figure 5:**
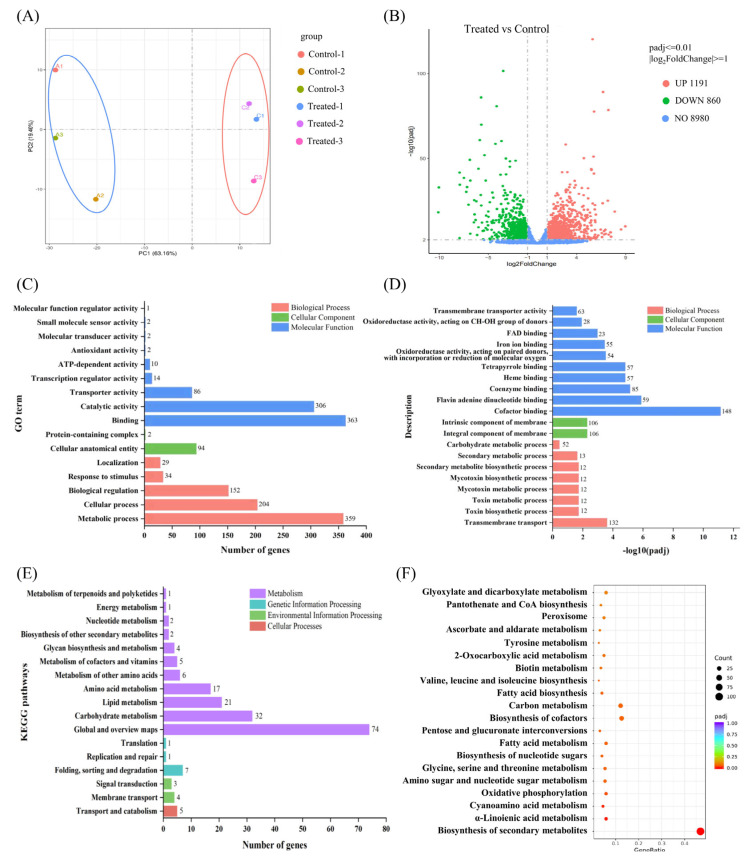
Transcriptome analysis of *V. mali* with genistein treatment. (**A**) PCA assays of control and treated group samples; (**B**) DEGs obtained by transcriptome assays; (**C**,**D**) GO pathway enrichment analysis based on DEGs; (**E**,**F**) KEGG pathway enrichment analysis based on DEGs.

To further investigate the changes in the transcription of genistein in *V. mali*, we collected nine samples, including untreated and treated mycelia, with three biological replicates (Figure 5A). A total of six samples were used to produce RNA-Seq reads using the Illumina NovaSeq platform (Table 1). Among all the sequencing samples, approximately seven Gb of raw reads and (obtained reads) were obtained. To ensure the quality and reliability of the data analysis, we filtered the raw data to obtain clean data, and the sequencing results from each sample had similar Q20 (>98.24%) and Q30 (>94.99%) scores and GC contents (56.59–57.02%). The sequencing error rate was only 0.02%, which was considerably lower than that of the normal condition (1%). The above data quality control results indicate that the high-quality clean data obtained can be used for subsequent analysis.

### 2.6. Differentially Expressed Genes (DEGs) Expression Analysis

Reference genome and gene model annotation files were downloaded directly from the genome website. Using HISAT2 v2.0.5, the clean reads were aligned to the reference genome (PRJNA268126) based on the downloaded files. The results of read mapping demonstrated that all the sequencing data were of high quality because the proportion of reads (≥95.86%) aligned to the unique site of the reference genome exceeded 70%, and the number of reads (≤0.54%) aligned to numerous positions did not exceed 10% (Table 2). As a result, reads that were aligned to the reference genome’s specific location were employed for subsequent quantitative data analysis.

Before the quantitative analysis of gene expression levels, the number of reads covered by each gene (including newly predicted genes) from beginning to end was calculated based on the positional information of the gene alignment on the reference genome. The majority of the filtered reads were noncontrasting pairs, reads with alignment quality values less than 10, and reads that were matched to several different genomic regions. The gene expression level of an RNA sequence is typically reported using the FPKM value rather than the read count because of the impact of sequencing depth and gene length. FPKM mapping resulted in several corrections to the gene length and sequencing depth. We then displayed the distribution of gene expression levels in various samples after determining the FPKM value of each gene in each sample. Moreover, the correlation coefficients of the intragroup and intergroup samples were calculated based on the FPKM values of all the genes in each sample, and a heatmap was drawn to visually display the differences and repetitions of the intergroup samples. In addition, all of the intragroup biological duplicate R^2^ values in this investigation were greater than 0.8 and greater than the R^2^ values of the intergroup biological samples. These results set the stage for later differential gene analysis to produce more trustworthy data. After the quantification of gene expression levels, we screened for genes whose expression levels significantly differed among the three groups of samples.

### 2.7. Gene Ontology (GO) Term and Kyoto Encyclopedia of Genes and Genomes (KEGG) Functional Enrichment Analyses of DEGs

To further investigate the variations in transcript abundance and expression patterns of genistein compared with those of *V. mali*, the Illumina RNA-Seq reads were mapped to the reference genome to determine the expression levels of the transcripts using the RPKM value. DESeq2 R package (1.20.0) was used to carry out DEG analysis based on the RNA-Seq data. A total of 923 DEGs (treated group vs. control group), including 618 upregulated genes and 305 downregulated genes in the treatment group (Figure 5B), were subsequently subjected to functional gene analysis for further exploration.

All DEGs were subjected to BLAST searches against the GO and KEGG databases using the clusterProfiler R package (3.8.1) to define their molecular functions. For the GO classification analysis, all DEGs were assigned to three main GO functional categories, namely, biological process (BP), cellular component (CC), and molecular function (MF), and were categorized into 20 main terms. As depicted in Figure 5C, the most prevalent subcategories within each of these three primary functional categories are distinctly outlined and presented separately. Among them, “binding”, “metabolic process”, and “catalytic activity” were the dominant terms. In the MF category, notable enrichment was observed among DEGs involved in “binding” (363 genes) and “catalytic activity” (306 genes). A total of 94 genes were enriched in the CC category “cellular anatomical entity”. “Metabolic process” (359 genes), “cellular processes” (204 genes), and “biological regulation” (152 genes) were the main terms within the BP category. We subsequently analyzed all the DEGs and displayed the GO terms with the most significant enrichment in each category, as shown in Figure 5D. In the MF category, the enrichment of DEGs involved in “cofactor binding”, “flavin adenine dinucleotide binding”, “coenzyme binding”, “heme binding”, and “tetrapyrrole binding” was most significant. Moreover, 54 and 28 genes were involved in “oxidoreductase activity, acting on paired donors, with incorporation or reduction of molecular oxygen” and “oxidoreductase activity, acting on the CH-OH group of donors”, respectively, which may be related to the oxidation and antioxidant processes within the mycelium. In addition, 63 genes related to “transmembrane transporter activity” were significantly enriched, suggesting that the integrity of *V. mali* cell membranes may be compromised. This speculation was also supported in the other two categories, which was consistent with the TEM results regarding cell membrane damage. For the CC category, 106 genes were actively involved in the “integral component of the membrane”, whereas 106 genes participated in the “intrinsic component of the membrane”. In the context of BP function, notable enrichment was observed among all DEGs involved in “transmembrane transport”. Therefore, we focused on the mechanism of cell membrane damage caused by genistein in subsequent research. Moreover, DEGs involved in the “toxin biosynthetic process”, “toxin metabolism process”, “mycotoxin metabolic process”, “mycotoxin biosynthetic process”, “secondary metabolite biosynthetic process”, and “secondary metabolic process” were significantly enriched, suggesting that genistein may exert its antifungal mechanism by modulating these genes, thereby mitigating the production of detrimental substances or metabolites by *V. mali*. The above findings indicate that genistein may affect transmembrane transport, redox function, and metabolic processes, which affect normal substance exchange, energy conversion within biological membranes, and metabolic activities.

KEGG pathway enrichment analysis of DEGs is a way to indicate whether there are significant differences in certain pathways. According to the functional distribution of the DEGs, they were distributed into three main groups: metabolism, genetic information processing, environmental information processing, and cellular processes. (Figure 5E). All DEGs were annotated to 74 KEGG pathways, and metabolic processes were dominant. As shown in Figure 5F, KEGG enrichment analysis revealed that the DEGs were enriched in 20 pathways. “Biosynthesis of secondary metabolites” stands as the preeminent enriched pathway, with a pivotal role in the biological process category, involving a total of 104 genes. Concurrently, “fatty acid metabolism”, “fatty acid biosynthesis”, and “oxidative phosphorylation” were significantly enriched, incorporating a substantial number of genes into their respective pathways.

### 2.8. Effects of Genistein on the Cell Membrane System of V. mali

**Figure 6:**
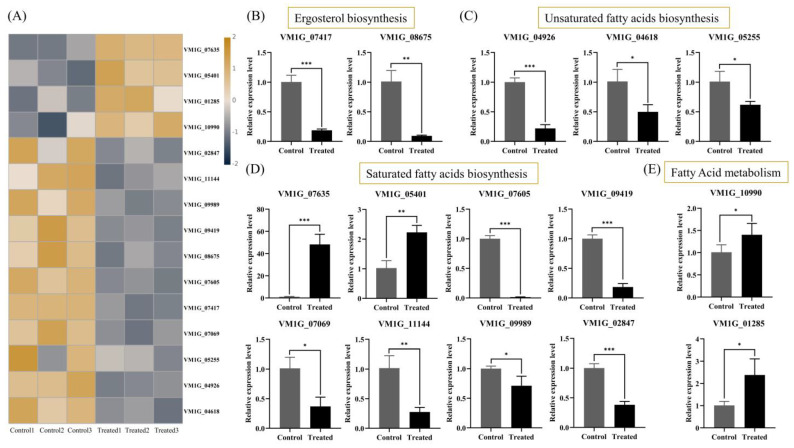
The regulation of the cell membrane system of *V. mali* by genistein. (**A**) A total of 15 DEGs were detected in the regulation cell membrane system by transcriptome. (**B**–**E**) Gene expression levels of 15 DEGs verified by qRT-PCR. * *p* < 0.05, ** *p* < 0.01, *** *p* < 0.001.

Based on the results of the transcriptome analysis, our treatment with genistein resulted in a decrease in the expression levels of multiple genes involved in fungal cell membrane stability. Among them, two genes were involved in the biosynthesis of ergosterol, three genes were involved in the biosynthesis of unsaturated fatty acids, six genes were involved in the biosynthesis of saturated fatty acids, and nine genes were involved in the biosynthesis of lipid metabolism (Figure 6A). The quantitative real-time reverse transcription PCR (qRT-PCR) validation results revealed that the expression levels of 15 genes, including VM1G_04618, VM1G_04926, VM1G_05255, VM1G_07069, VM1G_07417, VM1G_07605, VM1G_08675, VM1G_09419, VM1G_09989, VM1G_11144, VM1G_07635, VM1G_05401, VM1G_10990, VM1G_02847, and VM1G_01285 were consistent with the transcriptome sequencing results (Figure 6B,E). Among them, a total of 13 DEGs were associated with lipid peroxidation, with nine genes downregulated and four genes upregulated. Moreover, genes related to the biosynthesis of unsaturated fatty acids and saturated fatty acids were also differentially regulated. A decrease in the ratio of unsaturated fatty acids to saturated fatty acids affects the fluidity and structural stability of the plasma membrane [14]. These results further indicate that the structure and function of the cell membrane may be abnormal. Therefore, we speculate that changes in the proportion of fatty acids lead to lipid peroxidation, which in turn inhibits the normal growth of hyphae. In addition, ergosterol is the main component of the cell membrane. Research has demonstrated that citral inhibits *Penicillium digitatum* by disrupting the biosynthesis of ergosterol [15]. In our study, two genes involved in ergosterol biosynthesis were significantly downregulated in the treatment group. In summary, we speculate that genistein may exert antifungal activity by disrupting the cell membrane system of *V. mali*. However, TEM analysis revealed that the hyphal cell wall incurred damage. Future research should investigate the molecular mechanisms by which genistein inhibits the plant-derived fungus *V. mali*, with particular attention paid to aspects such as cell wall damage.

## 3. Discussion

*V. mali* is a necrotrophic fungus causing AVC, which limits apple production in China. This weakly parasitic fungus is characterized by its ability to establish a latent infection. According to reports, after infecting host tissue, *V. mali* mycelium can remain dormant at the wound site and survive in the xylem for many years [16]. In general, the various wounds caused by frostbite, sun exposure, trimming of the ends, and other mechanical injuries are the main pathways leading to *V. mali* infection in apple trees. This pathogen primarily damages apple trunks, branches, scaffolds, and fruits, significantly impacting fruit yield and quality, thereby inflicting substantial economic losses on the apple industry.

At present, the prevention and control of this disease mainly rely on measures such as scraping off the scar and applying chemical agents or spraying chemical agents across the entire tree [17]. However, *V. mali* may infect the woody parts of apple branches from cuts and other wounds and remain dormant for a long time, making it difficult for chemical agents to directly kill the pathogens [18]. In addition, long-term use of chemical pesticides may cause pathogenic fungi to develop drug resistance, leading to the deterioration of orchard ecological environments [19]. At the same time, chemical pesticides may cause a decline in fruit quality and even lead to food safety issues.

Plant-derived pesticides are safe, efficient, and residue-free “green pesticides” extracted from plants. Compared with chemical pesticides, they are easily degradable, safe for nontarget organisms, have low resistance development in harmful organisms, and exhibit a special mode of action [20]. In our previous research, we accidentally discovered that genistein has the potential to inhibit *V. mali* [21]. Genistein (4′,5,7-trihydroxyisoflavone) is a secondary metabolite of isoflavone predominantly sourced from leguminous plants. It has been identified in a variety of plant species [22,23,24,25,26,27,28,29,30], with a notable presence in *Glycine max* [31]. Based on data from the Web of Science (WOS) database, over 2200 studies in the past five years have documented various biological activities of genistein. The majority of these investigations have concentrated on its potential roles in the prevention and treatment of cancer [32], cardiovascular diseases [33], neurological disorders [34], and diabetes [35]. 

As a typical representative of plant estrogens, genistein is essentially an endocrine disruptor. At present, leguminous ingredients and soy products are considered the main sources of human intake of genistein. However, improper intake of genistein may disrupt hormone balance in the human body, especially estrogen receptor signaling, leading to a range of health problems such as reproductive dysfunction and breast cancer. Hu carried out a risk assessment on the potential harm of genistein intake to human breast health and found that improper intake of genistein had a certain negative impact on breast tissue development, breast cancer course development, and chemosensitivity [36]. At the same time, the authors also estimated that genistein intake in postmenopausal breast cancer patients poses a greater potential risk of harm to disease progression and chemotherapy treatment process; additionally, the mean risk quotient for minors on the Chinese mainland affected by dietary genistein intake on breast development was 45.56, with a 90% confidence interval range of 7.80–126.70. This shows that genistein is very harmful to the health of postmenopausal breast cancer patients and young people. In another study, the authors found that treatment with a dose of 100 mg/kg genistein in neonatal mice severely disrupted the development of ovarian and uterine structures, which is related to its inhibition of cell proliferation activity in ovarian and uterine tissues [37]. In view of the current consumption trends of legumes and the rising incidence of breast cancer and other related diseases in recent years, greater attention should be paid to the safety of legumes, related products, and even healthcare products for at-risk populations. In addition, Rocha also studied the impact of endocrine disruptor compounds on the environment. Due to signs of pollution at the mouth of the Mondego River, Rocha et al. measured the levels of endocrine disruptors in the water samples [38]. However, the results showed that more biological and toxicological analysis is still needed to confirm the estrogenic contributions of these plant estrogens and other compounds in the estuarine environment in order to fully understand their effects on local animal populations and humans.

To the best of our knowledge, there have been no reports on the inhibitory effects and mechanisms of genistein on plant pathogens. In the present study, the growth inhibition rate of *V. mali* treated with 10 µg/mL genistein reached 42.36 ± 3.22%, indicating that genistein has significant antifungal activity. SEM analysis demonstrated that genistein induced notable alterations in the structure of *V. mali*, encompassing mycelial contraction, distortion, deformity, collapse, as well as irregular protrusions. Furthermore, TEM analysis unveiled the leakage of cellular contents, blurred cell walls, membrane ruptures, and abnormalities in organelles. Through GO enrichment analysis of differentially expressed genes, we found that multiple genes related to cell membrane function were enriched in “integral component of membrane”, “intrinsic component of membrane”, “transmembrane transport”, and “transmembrane transporter activity”. Therefore, we focused on the mechanism of cell membrane damage by genistein in subsequent research. The dynamic balance between lipid peroxidation and oxygen-free radical reactions in fungal cells is maintained to support normal metabolic processes. Once the equilibrium between the two is disturbed, the permeability of the cell membrane rises, leading to cellular damage [11]. After treatment with 10 µg/mL genistein, green fluorescence in mycelia was significantly higher than that in the untreated group. This suggested that genistein caused ROS accumulation in mycelia. Research has shown that CAT, POD, and SOD are the main protective enzymes in fungi, which resist oxidative stress damage to tissues by clearing reactive oxygen species in the fungal body. In this study, after treatment with genistein, the activity of the antioxidant protective enzyme SOD decreased. In contrast, the activities of CAT and POD increased, leading to an imbalance between oxidation and antioxidant processes in the mycelium, resulting in oxidative stress and promoting the accumulation of reactive oxygen species in the mycelium. Meanwhile, molecular docking results showed that genistein exhibited strong binding affinity with SOD(3K9S), POD(2 × 08), and CAT(8J4Q), which supported the above results. More importantly, based on the results of molecular docking, we analyzed and speculated that genistein might adapt and stably bind to the active pockets of the above proteins. This would reduce the flexibility and root mean square deviation of the proteins, alter their dynamic and thermodynamic conformations, and thereby increase the stability of the proteins [39,40]. These interactions are crucial for the stability of proteins and compounds and can significantly affect their biological functions [41,42].

The sudden increase in ROS content within the mycelium is an important indicator of lipid peroxidation in biological membranes [43]. Therefore, we focused on differentially expressed genes related to lipid peroxidation and found that 13 differentially expressed genes were associated with lipid peroxidation, with nine genes downregulated and four genes upregulated. Meanwhile, genes related to unsaturated fatty acids and saturated fatty acids are also regulated. The decrease in the ratio of unsaturated fatty acids to saturated fatty acids will affect the fluidity and structural stability of the plasma membrane. These results further indicate that the structure and function of the cell membrane may be abnormal. Therefore, we speculate that changes in the proportion of fatty acids lead to lipid peroxidation, which in turn inhibits the normal growth of hyphae. In addition, ergosterol, as the main component of the cell membrane, has significantly downregulated two genes involved in its biosynthesis. Therefore, we speculate that genistein may exert antifungal activity by triggering ROS-mediated lipid peroxidation, further disrupting the cell membrane system of *V. mali*. However, the 15 candidate DEGs we selected did not receive further experimental validation. Future research will aim to confirm these mechanisms at the molecular level.

## 4. Materials and Methods

### 4.1. Media, Pathogens and Reagents

Genistein (batch number: G1719012) was purchased from Aladdin Biochemical Technology Co., Ltd. (Shanghai, China). *V. mali* was provided by the Tobacco Research Institute of the Chinese Academy of Agricultural Sciences (Qingdao, China) [44]. Potato dextrose agar (PDA, batch number: G1719012) from Beijing Land Bridge Technology Co., Ltd. (Beijing, China) and DCFH-DA detection kits (batch number: D06IR2025SA) from Solarbio (Beijing, China) were used in this study. SOD (batch number: A001-3-1), CAT (batch number: A007-1-1), and POD (batch number: A084-3-1) kits were obtained from Nanjing Jiancheng Bioengineering Institute (Nanjing, China).

### 4.2. Growth Inhibition Assay of Genistein Against V. mali

The micro double-dilution method was used to determine the MIC of genistein that inhibited *V. mali*. The sterilized culture medium was added to a 96-well plate, and different concentrations of genistein (40, 20, 10, 5, 2.5, 1.25, 0.62, and 0.31 µg/mL) were added, followed by the addition of the fungal mixture [45]. Each group was analyzed in triplicate. Then, the 96-well plates were placed in a constant-temperature incubator at 25 °C for 20 h. The maximum concentration of medium without turbidity in a 96 well plate was recorded as the MIC value.

Using *V. mali* as the test strain, the effect of genistein on fungal hyphal growth was detected using the growth rate method [12]. Under sterile conditions, a 1:100 mixture of 10 µg/mL genistein and autoclaved PDA medium was poured into plates. An equal volume of 95% aqueous ethanol without genistein or other reagents was added to the control group. Each group was analyzed in triplicate. After the agar plates solidified, 9 mm long fungal cakes were obtained from the edges of single colonies, and the plates were inoculated and cultured in a 25 °C incubator. When the mycelia of the control group spread to the edge of the culture dish, the diameters of the fungal colonies were measured using the cross method. The growth inhibition rate of *V. mali* by genistein was calculated according to the following formula:

Inhibition (%) = [(average diameter of the control group − average diameter of the treatment group)/(average diameter of the control − 9)] × 100%

### 4.3. Preparation and Examination of Samples for SEM and TEM

For SEM analysis, *V. mali* was cultured on PDA medium supplemented with 10 µg/mL genistein in the dark at 25 °C for 3 days. Mycelia were selected, cut, and fixed in 3% aqueous glutaraldehyde for 4 h. Subsequently, they were washed six times with phosphate-buffered saline (0.1 M, pH = 6.8) for 20 min to remove the glutaraldehyde as thoroughly as possible and then dehydrated using graded aqueous ethanol solutions (30%, 50%, 70%, 80%, 90%, 95%, and 100%) for 30 min at each concentration. The mycelia were subsequently transferred to isoamyl acetate for 3 h, which made the mycelia fuller and prevented deformation or wrinkling. After the samples were dried at the critical point of carbon dioxide and sprayed with gold using an ion-sputtering instrument, they were observed and recorded using a SU8010 scanning electron microscope (HITACHI, Tokyo, Japan).

TEM analysis of the samples was performed using the sample processing and dehydration methods described above. After dehydration, the mycelial samples were embedded in epoxy resin and then cut into mycelial slices of approximately 70 nm by ultrathin sectioning. After double staining with uranyl acetate and lead citrate, the ultrastructure of untreated and treated mycelia was observed using an H-7650 transmission electron microscope (HITACHI, Tokyo, Japan).

### 4.4. Determination of the Intracellular ROS Content

To further verify the antifungal activity of genistein, which is based on the oxidative stress mechanism, the ROS level in mycelia was detected [46]. The ROS levels of the samples were measured using a reactive oxygen species assay kit (CA1410; Beijing Solarbio Science & Technology Co., Ltd., Beijing, China). The obtained *V. mali* mycelia were processed with DCFH-DA for 20 min at 28 °C. Mycelia were subsequently observed with a TE2000U fluorescence microscope (Nikon Ltd., Tokyo, Japan).

### 4.5. Determination of the Activities of Antioxidant Enzymes

*V. mali* mycelium was treated according to a previously described method. The SOD activity, POD activity, and CAT activity were measured using different commercial kits (A001-1, A084-3, A007-1, Nanjing Jiancheng Biotechnology Research Institute, China), respectively [12]. The structure of the compound was downloaded from the PubChem database and imported into ChemBio3D 14.0 software to adjust the spatial conformation of active ingredients and calculate the optimization of energy. After AutoDockTools 1.5.6 processing, the files were saved in pdbqt format [47]. The three-dimensional crystal structure of the target proteins, including SOD (PDB code: 3K9S, resolution: 1.55 Å), POD (PDB code: 2X08, resolution: 2.01 Å), and CAT (PDB code: 8J4Q, resolution: 2.10 Å), was downloaded from the PDB database (https://www.rcsb.org/ accessed on 23 September 2024). The water molecule and organic matter in the target protein were removed by Notepad2, and then the target protein was imported into AutoDockTools 1.5.6 for hydrogenation, charge distribution, and atomic type addition [48]. AutoDockVina was used for molecular docking, and the docking results were plotted using Pymol 2.6.1 [49].

### 4.6. RNA Extraction and Sequencing

For RNA-Seq analysis, *V. mali* was treated with genistein using the same methods as those described above but with slight modifications. Briefly, mycelia (0.3 g) on genistein-treated (treated group) and control plates (control group) were also collected and transferred to sterile centrifuge tubes, quickly frozen in liquid nitrogen, and stored at −80 °C for subsequent total RNA extraction. Both the treatment and control experiments were performed in triplicate. The total RNA of each sample was extracted using TRIzol reagent (Life Technologies, Carlsbad, CA, USA) on dry ice and processed following the protocol provided by the manufacturer. The integrity of the RNA was assessed using an RNA Nano 6000 Assay Kit on a Bioanalyzer 2100 system (Agilent Technologies, California, CA, USA). mRNA was purified from total RNA using poly-T oligo-attached magnetic beads. An AMPure XP system (Beckman Coulter, Beverly, CA, USA) was used to purify the library fragments to select cDNA fragments that were preferentially 370–420 bp in length. This system was also used to purify the PCR products. The Agilent Bioanalyzer 2100 system was subsequently used for quality assessment of the cDNA library. Clustering of the index-coded samples was performed on a cBot Cluster Generation System using the TruSeg PE Cluster Kit v3-cBot-HS (Illumina) according to the manufacturer’s instructions. After cluster generation, the library preparations were sequenced on the Illumina NovaSeq platform by Beijing Nuohe Zhiyuan Technology Co., Ltd. (Beijing, China).

### 4.7. Analysis of Sequencing Data

After processing raw data (raw reads) in fastq format through in-house Perl scripts, clean data (clean reads) were obtained by removing reads containing adapters, reads containing poly-N runs, and low-quality reads from the raw data. Moreover, the Q20 and Q30 scores and the GC content of the clean data were calculated. All the downstream analyses were based on high-quality, clean data. HISAT2 v2.0.5 was used to construct an index of the reference genome and align paired-end clean reads to it. Then, the HISAT2 mapping tool was used to generate a database of splice junctions with better mapping results [50].

Feature Counts vl.5.0-p3 was used to count the number of reads mapped to each gene. The gene expression levels were subsequently calculated and normalized to the FPKM values based on the lengths of the genes and reads [51,52,53]. Principal component analysis is also commonly used to evaluate intergroup differences and intragroup sample duplication. PCA uses linear algebra to reduce dimensionality and extract principal components from tens of thousands of gene variables. Differential gene expression analysis of the three groups was performed using the DESeq2R package (1.20.0). To control the false discovery rate, Benjamini and Hochberg’s approach was used to adjust the resulting *p* values [54,55,56,57]. Genes with *p* values < 0.05 and absolute fold changes ≥1 were considered differentially expressed. GO enrichment analysis of DEGs was implemented by the clusterProfiler R package, in which gene length bias was corrected. We used the clusterProfiler R package to test the statistical enrichment of DEGs in KEGG pathways (https://www.genome.jp/kegg/ (accessed on 2 March 2023)).

### 4.8. qRT-PCR Analysis

The expression profiles of ten randomly selected DEGs were analyzed by qRT-PCR to confirm the accuracy of the Illumina RNA-Seq data. The SPARKeasy Improved Tissue/Cell RNA Kit, SPARKscriptⅡRT Plus Kit (With gDNAEraser), and 2 × SYBR Green qPCR Mix Kit (Shandong Sparkjade Biotechnology Co., Ltd., Jinan, China) were used for total RNA preparation, reverse transcription and qRT-PCR analysis of the DEGs. The expression of the α-actin gene was used as the internal standard. The primers were designed, and their sequences are listed in Table 3. The PCR program was as follows: initial denaturation at 95 °C for 3 min, followed by 40 cycles of denaturation at 95 °C for 10 s, annealing at 55 °C for 30 s, and extension at 72 °C for 30 s (Jena Analytical Instruments GmbH, Jena, Germany). The relative expression levels of selected genes were analyzed using the 2^−ΔΔCt^ method.

### 4.9. Statistical Analysis

To compare the differences between groups, an independent sample *t*-test was conducted. Each group consisted of three replicate experiments to ensure the reliability of the results. The following symbols are used to indicate significance levels: * represents *p* < 0.05; ** represents *p* < 0.01; and *** represents *p* < 0.001.

## 5. Conclusions

**Figure 7:**
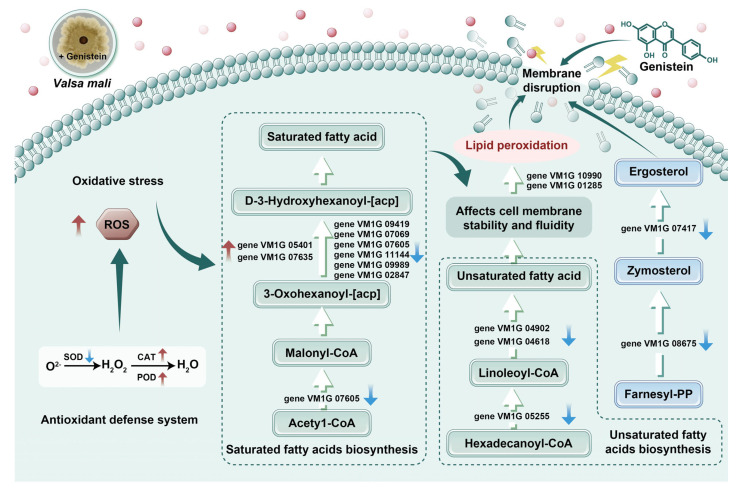
The mechanism of genistein inhibiting *V. mali* growth through ROS-mediated lipid peroxidation.

This study provides a preliminary investigation into the inhibitory activity and mechanism of genistein on *V. mali*. This study revealed that genistein induced cell membrane damage in *V. mali*, as evidenced by TEM of the hyphae and corroborated by the transcriptome sequencing data. Additionally, genistein promoted the accumulation of ROS, leading to oxidative stress and the activation of the antioxidant defense system. This was specifically observed as a decrease in SOD activity and an increase in CAT and POD activities. Moreover, genistein was implicated in the regulation of the biosynthesis of unsaturated (three DEGs) and saturated fatty acids (nine DEGs), potentially altering the fatty acid composition and ultimately resulting in lipid peroxidation. Furthermore, thirteen genes associated with fatty acid metabolism were identified that may be linked to membrane lipid peroxidation, resulting in oxidative stress damage to the mycelia and ultimately inhibiting their growth (Figure 7). In conclusion, this study is the first to demonstrate that genistein may exert antifungal activity by disrupting the cell membrane system of *V. mali*.

## Figures and Tables

**Table 1:** Quality assessment of sequencing data.

Samples	Control-1	Control-2	Control-3	Treated-1	Treated-2	Treated-3
Raw reads	46,768,938	47,448,424	48,968,902	46,731,424	45,432,282	45,201,716
Raw bases	7.02 G	7.12 G	7.35 G	7.01 G	6.81 G	6.78 G
Clean reads	45,370,436	46,266,524	47,631,958	45,241,510	44,104,334	44,120,272
Clean reads/raw reads (%)	97.01%	97.51%	97.27%	96.81%	97.08%	97.61%
Clean bases	6.81 G	6.94 G	7.14 G	6.79 G	6.62 G	6.62 G
Error rate	0.02	0.02	0.02	0.02	0.02	0.02
Q20	98.53	98.34	98.42	98.39	98.36	98.36
Q30	95.79	95.25	95.47	95.44	95.33	95.32
GC pct	56.73	56.87	57.02	56.59	56.62	56.71

Note: Raw reads: the number of reads in the raw data; raw bases: the base number of raw data (raw base = raw reads × 150 bp); clean reads: the number of reads filtered from the raw data; clean bases: the number of bases filtered from the raw data (clean base = clean reads × 150 bp); error rate: overall sequencing error rate of data; Q20: percentage of bases with Phred values greater than 20 in the total number of bases; Q30: percentage of bases with Phred values greater than 30 in the total number of bases; GC pct: the percentage of G and C bases in clean reads.

**Table 2:** Reads mapping to the reference genome.

Samples	Total Reads	Total Map	Unique Map	Multi Map
Control-1	45,370,436	43,541,348 (95.97%)	43,327,458 (95.5%)	213,890 (0.47%)
Control-2	46,266,524	44,370,961 (95.9%)	44,121,004 (95.36%)	249,957 (0.54%)
Control-3	47,631,958	45,658,834 (95.86%)	45,424,635 (95.37%)	234,199 (0.49%)
Treated-1	45,241,510	43,476,439 (96.1%)	43,277,906 (95.66%)	198,533 (0.44%)
Treated-2	44,104,334	42,428,393 (96.2%)	42,239,746 (95.77%)	188,647 (0.43%)
Treated-3	44,120,272	42,441,929 (96.2%)	42,227,638 (95.71%)	214,291 (0.49%)

Note: Total reads: the number of clean reads of sequencing data after quality control; total map: the number and percentage of reads aligned to the genome; unique map: the number and percentage of reads aligned to the unique position of the reference genome (used for subsequent quantitative data analysis of reads); multi map: the number and percentage of reads aligned to multiple positions in the reference genome.

**Table 3:** Primers for qRT-PCR.

Genes Name	Primers (5′ to 3′)
*G6PDH* [58]	F: TCAGAACAAGTTCGAGGGCGACAA
	R: TGAGGGCAATAGAGGGCTTGTTCA
VM1G_04618	F: AGCTTTCCGGTCATTATCACA
	R: GTCCGAAGTCAATGATCCACA
VM1G_04926	F: AGCTCCTCTCGGTCGTGCTCA
	R: CACACCCACGGCCACGTCGTC
VM1G_05255	F: AAAGGCTCTTCTTGTCGTCCA
	R: TTTGCTGTCCTGCTCCTCGTC
VM1G_07069	F: ACACAACTCGCCATATCCCA
	R: TCCTCTTTGGATTCGACGATG
VM1G_07417	F: TGATTTCATGGACCTGCCGTT
	R: TAGATCTGGCTGTACACGCTCT
VM1G_07065	F: ACCATCCTGACATTGAGCCTA
	R: AACCTGCCCCTTTTGTACCAC
VM1G_08675	F: CTCACGATACCCGTATTTGGC
	R: AATCAAGGGAACATGCGACT
VM1G_09419	F: CGTGTTGGTCTTCCCATTGACA
	R: AGAACCTTGCCATTGATCCAC
VM1G_09989	F: CCGAGACACATCTACATGCAC
	R: ATATCATGCGGCCAATTCCC
VM1G_11144	F: GATACGTCTTGATGCACCGAT
	R: CTTTCCCAGTTGTCCGCTTG
VM1G_05401	F: AATGTATCACCGCAAAATCCG
	R: GTACTCTTGACTCAGCGACCA
VM1G_07635	F: GCGGTCCTTCCCTACCTCC
	R: CTACTCCCCTGGATAGCGTCT

## Data Availability

The data will be made available upon request.
